# Supplementary material for: Global daily 1 km land surface precipitation based on cloud cover-informed downscaling
Source: Sci Data. 2021 Nov 26;8:307. doi: 10.1038/s41597-021-01084-6 (PMC8626457; doi:10.1038/s41597-021-01084-6)
Supplement: Supplementary file 1 — Supplementary Figure 1 [file 41597_2021_1084_MOESM1_ESM.pdf]

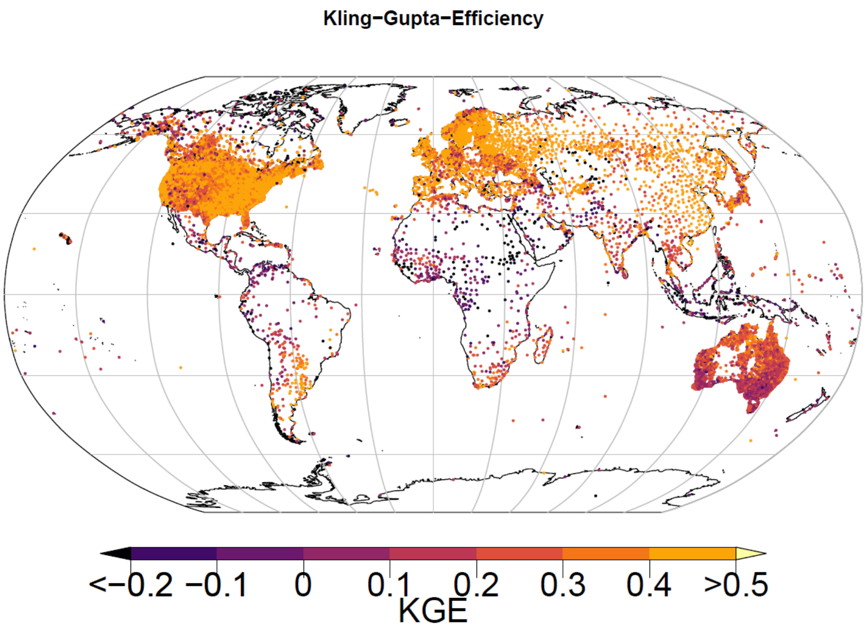

Supplementary Figure 1 | Kling-Gupta efficiency (KGE) values derived from a comparison of CHELSA_EarthEnv with GHCN-D. Data is based on 58,071 stations for the time period 2003 to 2016.
